# A Novel Time-Resolved Fluorescence Resonance Energy Transfer Assay for the Discovery of Small-Molecule Inhibitors of HIV-1 Tat-Regulated Transcription

**DOI:** 10.3390/ijms24119139

**Published:** 2023-05-23

**Authors:** Young Hyun Shin, Dong-Eun Kim, Kyung Lee Yu, Chul Min Park, Hong Gi Kim, Kyung-Chang Kim, Songmee Bae, Cheol-Hee Yoon

**Affiliations:** 1 Division of Chronic Viral Diseases, Center for Emerging Virus Research, Korea National Institute of Health, 187 Osongsaengmyeong 2-ro, Cheongju 363951, Republic of Korea; yhshin80@korea.kr (Y.H.S.); dongeunkim@korea.kr (D.-E.K.); smashing20@korea.kr (K.L.Y.); joytodeath@korea.kr (K.-C.K.); somgmee@korea.kr (S.B.); 2 Department for Convergent Research of Emerging Virus Infection, Korea Research Institute of Chemical Technology, 141 Gajeong-ro, Daejeon 34114, Republic of Korea; parkcm@krict.re.kr (C.M.P.); tenork@krict.re.kr (H.G.K.)

**Keywords:** Tat-TAR RNA interaction, time-resolved fluorescence resonance energy transfer, small-molecule inhibitor, high-throughput screening, europium cryptate, HIV-1 replication

## Abstract

Human immunodeficiency virus-1 (HIV-1) transactivator (Tat)-mediated transcription is essential for HIV-1 replication. It is determined by the interaction between Tat and transactivation response (TAR) RNA, a highly conserved process representing a prominent therapeutic target against HIV-1 replication. However, owing to the limitations of current high-throughput screening (HTS) assays, no drug that disrupts the Tat-TAR RNA interaction has been uncovered yet. We designed a homogenous (mix-and-read) time-resolved fluorescence resonance energy transfer (TR-FRET) assay using europium cryptate as a fluorescence donor. It was optimized by evaluating different probing systems for Tat-derived peptides or TAR RNA. The specificity of the optimal assay was validated by mutants of the Tat-derived peptides and TAR RNA fragment, individually and by competitive inhibition with known TAR RNA-binding peptides. The assay generated a constant Tat-TAR RNA interaction signal, discriminating the compounds that disrupted the interaction. Combined with a functional assay, the TR-FRET assay identified two small molecules (460-G06 and 463-H08) capable of inhibiting Tat activity and HIV-1 infection from a large-scale compound library. The simplicity, ease of operation, and rapidity of our assay render it suitable for HTS to identify Tat-TAR RNA interaction inhibitors. The identified compounds may also act as potent molecular scaffolds for developing a new HIV-1 drug class.

## 1. Introduction

Human immunodeficiency virus-1 (HIV-1), the causative agent of acquired immunodeficiency syndrome (AIDS), is a hazardous pathogen that threatens global public health. In 2020, approximately 37.7 million people were living with HIV and 0.68 million died from AIDS-related diseases [1]. Thirty species of anti-retroviral drugs (ARVs) have been developed that largely contribute to extending a patient’s life span by interrupting essential viral replication steps such as attachment, reverse transcription, integration, and maturation. However, poor therapeutic effects are sometimes associated with drug resistance mutations and toxicity during long-term treatment because the current ARVs cannot eliminate latently infected cells, termed reservoirs. Therefore, developing new small-molecule inhibitors that are capable of precisely interrupting viral replication is necessary. Most therapeutic drugs, including ARVs, have been developed as small molecules because of their beneficial properties, thus enabling multiple applications in drug discovery, such as permeability across biological barriers, target selectivity, metabolic stability, and/or ease of accessibility for chemical synthesis [2].

HIV-1 transcription is a highly organized process that is tightly controlled by HIV-1 transactivator (Tat), a small 14 kDa protein consisting of 86–101 amino acids. HIV-1 transcription is naturally initiated in the viral long terminal repeat (LTR) by host transcriptional factors, such as nuclear factor κB (NF-κB), Sp1, and nuclear factor of activated T cells (NFAT) [3,4,5,6]; however, transcription is not achieved efficiently without the Tat protein. The expressed Tat protein immediately binds to transactivation-responsive element (TAR) RNA at the 5′ ends of all nascent viral transcripts and simultaneously recruits the super elongation complex (SEC) containing positive transcription elongation factor b (P-TEFb), facilitating viral transcriptional elongation [7,8]. The Tat protein contains an arginine-rich motif (ARM; amino acids 49–57) for binding to TAR RNA and nuclear localization. A consensus sequence of R/KxxRRxRR in this ARM is critical for TAR RNA recognition [9,10,11]. The minimal region from nt +19 to +43 in the TAR RNA element is sufficient for Tat-induced transcriptional activity [9]. In addition, the trinucleotide bulge (U23-C24-U25) in TAR RNA is crucial for Tat recognition, besides which several nucleotides positioned at +38, +40, and near-bulge contribute to Tat interaction [12,13].

The Tat-TAR RNA interaction has been considered a therapeutic target against HIV-1 infection due to being a distinct process separate from the host cellular system [14,15,16]. Therefore, numerous studies have attempted to identify an agent targeting Tat-dependent viral transcription using diverse screening approaches, but a therapeutic compound disrupting the Tat-TAR RNA interaction has not yet been discovered. Computational virtual screening based on in silico structural docking was used for high-throughput screening (HTS) [17,18,19,20], and a nuclear magnetic resonance (NMR) study using a TAR RNA ligand identified small probe molecules targeting TAR RNA [20,21]. Although these methods were helpful in predicting compound structures that could bind to Tat, TAR RNA, or both, further efforts were needed to confirm Tat-TAR RNA dissociation by the identified compounds. Biochemical approaches such as electrophoretic mobility shift assay (EMSA), filter binding assay, and/or scintillation proximity assay (SPA) are widely used to characterize the interaction between proteins and nucleic acids [22,23,24,25]; however, these methods require laborious work, including immobilization of TAR RNA or Tat proteins on membranes or beads, washing, and/or isotope-labeling processes. Accordingly, these methods are generally used in small-scale screenings or confirmatory tests of selected compounds. The fluorescence resonance energy transfer (FRET) technique has often been used to confirm whether the TAR RNA-binding compounds identified during primary screening methods, such as NMR/computational prediction and colorimetric screening, disrupt the Tat-TAR RNA interaction [18,26,27]. Although the FRET technique has been widely used to determine the binding affinities of two interacting partners, it is not appropriate for highly sensitive measurements because of the high level of background noise derived from the scattered excitation light of the donor fluorophore [28,29]. The time-resolved FRET (TR-FRET) technology is based on the use of europium (Eu) chelate (absorption peak range, approximately 320–340 nm; emission peak range, approximately 610–620 nm) [30] and cyanine 5 (or allophycocyanin, or ULight) as the donor and acceptor, respectively; it is an advanced version of the FRET approach that allows for homogeneous (mix-and-read) phase assays with reduced background fluorescence [31,32,33,34].

In the present study, we aimed to design an assay using the TR-FRET technology to characterize the Tat protein-TAR RNA interaction. The assay sensitively and successfully detected the inhibitors of the Tat-TAR RNA interaction and showed a stable TR-FRET signal applicable to HTS in a 384-well microplate. Moreover, the assay successfully screened novel inhibitory compounds from a 39 360-compound library that disrupted the Tat-TAR RNA interaction, which efficiently inhibited the activity of both Tat and HIV-1.

## 2. Results

### 2.1. Design of the TR-FRET Assays and Selection of a Suitable Pair of Components

**Figure 1:**
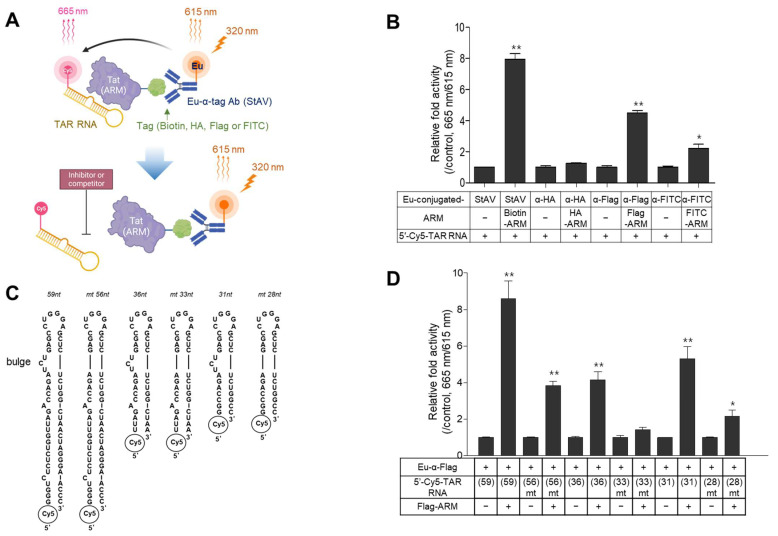
Configuration of the established time-resolved fluorescence resonance energy transfer (TR-FRET) assay and comparison of the tagging system and trans-activating response (TAR)-RNA fragments. (**A**) Schematic illustration of the TR-FRET assay to detect the interaction of 5′-Cy5-labeled TAR RNA and Tat-derived peptide (arginine-rich motif, ARM) coupled with Eu-conjugated α-tagged antibody. When Tat (ARM) and TAR RNA interact, the fluorescence at 615 nm transferred from Eu excited at 320 nm excites 5′-Cy5-labeled TAR RNA, followed by the emission of a fluorescent signal at 665 nm, which is disrupted in the presence of an inhibitor for Tat (ARM)-TAR RNA interaction. (**B**) Twenty nanomoles of Tat (ARM) tagged with various epitopes were incubated with the same nanomolar 5′-Cy5-TAR RNA fragment (31 nt) for 30 min. Subsequently, 5 nM of Eu-conjugated recognizer was added to the reaction, incubated further for 30 min, and then detected as described in the Materials and Methods section. The interaction signals are expressed as a relative fold activity of (665 nm/615 nm) × 1000 compared to the control lacking each tagged Tat (ARM) peptide. (**C**) Structures and sequences of various lengths of wild-type (wt) TAR RNA fragments and bulge-free mutant (mt) forms. (**D**) Flag-tagged Tat (ARM) (20 nM) was reacted with the same nanomolar concentration of various 5′-Cy5-TAR RNA fragments. After incubation with the Eu-conjugated α-Flag antibody, the interaction signals were determined and expressed as relative fold activity compared with the control lacking each tagged Tat (ARM) peptide. All graphical data are presented as the mean ± SD (*n* = 3). All statistical analyses were performed using the Student’s *t*-tests (* *p* < 0.05, ** *p* < 0.01).

Initially, a simplified TR-FRET assay was designed to determine the interaction of Tat (ARM)-TAR RNA using 5′-cyanine 5 (Cy5)-labeled TAR RNA (31 nt) and Tat (ARM) tagged with either biotin, HA, FLAG, or FITC; this was based on size-minimalized analytes to focus on the essential biochemical relevance of the Tat-TAR RNA interaction. After Tat (ARM) binds to the TAR RNA, each Eu-conjugated antibody recognized the cognate tag on the Tat (ARM) peptide. When the reaction is irradiated with a 320 nm laser, the emissive energy from the donor Eu chelate is then transferred to the Cy5 dye-labeled to TAR RNA placed at a close distance (~10 nm), resulting in luminescence at 665 nm from Cy5 and 615 nm from Eu. A certain substance inhibiting the Tat-TAR RNA interaction causes the loss of luminescence in the sample at 665 nm emission (Figure 1A). To determine which Eu-conjugated recognizer (anti-Flag, -HA, FITC antibodies, or streptavidin) is appropriate for successfully establishing the TR-FRET assay, 20 nM of ARM tagged with each epitope and the same concentration of Cy5-labeled TAR RNA fragment were incubated with 5 nM of cognate Eu-conjugated recognizer. As shown in Figure 1B, the intensity of the TR-FRET signal was the highest in the reaction using biotinylated ARM paired with Eu-conjugated streptavidin (StAV). The Flag-tagged ARM also exhibited significantly enhanced signal intensity (fold activity of the signal-to-background ratio compared to the control lacking ARM, ~5). However, assays using anti-HA and FITC-tagged ARM exhibited less efficient time-resolved fluorescence at 665 nm (Figure 1B). Although an assay coupled with biotinylated ARM and Eu-conjugated StAV exhibited a high TR-FRET signal, the assay exhibited insufficient inhibition of the TR-FRET signal even in the five-thousand-fold excess of tag-free ARM as a competitor and L-22 as a known inhibitor (Supplementary Figure S1A). It showed high non-specific TR-FRET signals in the reactions with different lengths of bulge-free mutant (mt) TAR RNA (Supplementary Figure S1B).

Next, we designed another TR-FRET assay using ULight as an acceptor fluorophore, labeled with 5′-biotinylated TAR RNA (Supplementary Figure S2A). Although an assay combined with Flag-ARM exhibited the most potent TR-FRET signal in the presence of wild-type (wt) biotinylated TAR RNA, a non-specific signal was clearly detected even in the reaction with mt TAR RNA (Supplementary Figure S2B). A similar effect was observed in assays using other tagged ARMs (Supplementary Figure S2C,D). Another assay was designed using Eu-W8044-StAV as a donor fluorophore and ULight-conjugated-α tag antibodies (Supplementary Figure S3A). Unexpectedly, this assay also exhibited high non-specific signals in mt TAR RNA (Supplementary Figure S3B,C). These data indicated that the probing system using biotin-StAV largely obstructed the expression of specific TR-FRET signals for ARM-TAR RNA interaction under our experimental conditions.

Owing to the low specificity of TR-FRET assays using biotin-StAV, we employed a TR-FRET assay paired with 5′-Cy5-TAR RNA and Flag-ARM in our next study. To evaluate the specificity of the TR-FRET signal for ARM-TAR RNA interaction, different lengths of wt 5′-Cy5-TAR RNA or their bulge-free mt TAR RNA were used (Figure 1C). Flag-ARM (20 nM) was incubated with 20 nM of TAR RNA (59 nt), (36 nt), (31 nt), or each cognate bulge-free mt TAR RNA. As shown in Figure 1D, the TR-FRET signal with TAR RNA (59 nt) exhibited the highest signal, but the signal was robust, even in its mt form. TAR RNA (31 nt) resulted in a sufficient fluorescence signal, which was considerably decreased in its bulge-free mt form. The signal from TAR RNA (36 nt) was lesser compared with the use of other lengths (Figure 1D). These data indicated that the unnecessary stem regions of TAR RNA may affect the specificity of their interaction between the bulge and ARM.

### 2.2. Optimization of the TR-FRET Assay

**Figure 2:**
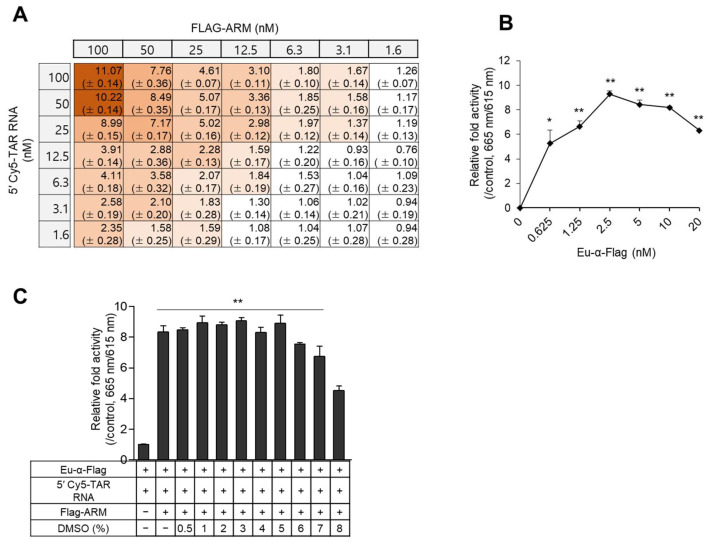
Titration for optimizing the TR-FRET assay. (**A**) 2D titration of Flag-ARM and TAR RNA. Two-fold serial dilutions of ARM and 5′-Cy5-TAR RNA started at 100 nM were incubated with 5 nM of Eu-conjugated α-Flag antibody in a 384 well-plate. The relative fold activity of TR-FRET signals is expressed as the mean ±SD (*n* = 3) compared with the controls lacking Flag-ARM or 5′-Cy5-TAR RNA. The intensity of the brown color indicates the extent of the signal. (**B**) Fifty nanomoles of Flag-ARM and 5′-Cy5-TAR RNA were incubated with two-fold serial dilution of Eu-conjugated α-Flag Ab. The relative fold activities are presented as the mean ± SD (*n* = 3) compared with the control lacking Eu-conjugated α-Flag Ab. (**C**) Fifty nanomoles of Flag-ARM and 5′-Cy5-TAR RNA were incubated with 2.5 nM of Eu-conjugated α-Flag antibody upon treatment with the indicated % of DMSO. The relative fold activities are expressed as the mean ± SD (*n* = 3) compared with the control lacking Flag- ARM. All statistical analyses were performed using Student’s *t*-tests (* *p* < 0.05 and ** *p* < 0.01).

To apply the assay to HTS, a minimal concentration of components capable of producing a potent signal is required for efficient and low-priced screening of several hundred thousand compounds in the library. We performed a two-dimensional (2D) titration assay with two-fold serial dilutions of 5′-Cy5-TAR RNA (31 nt) and Flag-ARM peptide with 5 nM of Eu-conjugated α-Flag antibody in a 384-well microplate. The TR-FRET signal gradually increased ~11-fold in a concentration-dependent manner until it reached 100 nM of analytes. As high concentrations of components are wasteful in HTS, 50 nM of each molecule exhibited a nearly saturated signal, which was used in subsequent experiments (Figure 2A). Next, a titration curve was performed for the Eu-conjugated antibody using 50 nM of ARM and TAR RNA. The maximum signal of 665 nm/615 nm was detected in the reaction using 2.5 nM of Eu-conjugated anti-Flag Ab, while the signal decreased at higher concentrations (Figure 2B). A dimethyl sulfoxide (DMSO) tolerance test was then performed to determine the maximum solvent limit. With increasing amounts of DMSO, the maximum TR-FRET signal decreased slightly; however, the signal became steady with up to 5% DMSO (Figure 2C).

### 2.3. Evaluation of the Assay

**Figure 3:**
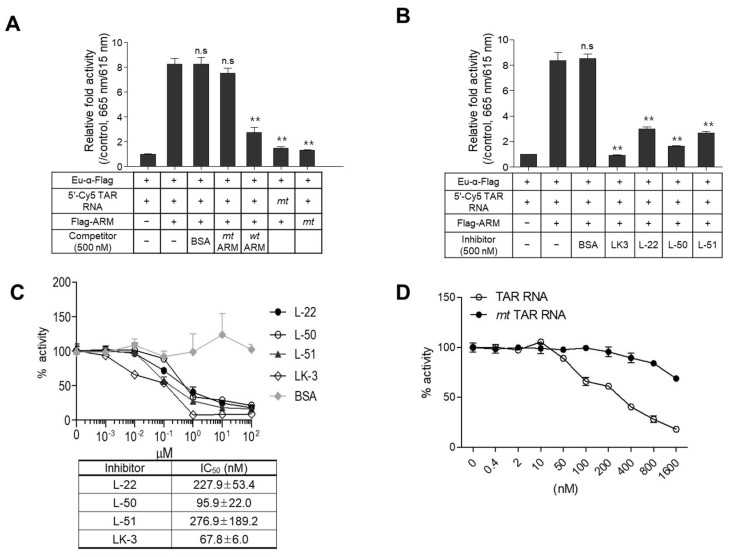
Specificity of the assay with known inhibitors. (**A**,**B**) Fifty nanomoles of wild-type (wt) Flag-tagged ARM and mutant (mt) ARM were incubated with wt 5′-Cy5-TAR RNA or mt 5′-Cy5-TAR RNA in the presence or absence of non-tagged wt ARM/mt ARM (**A**) and known inhibitors (**B**) upon treatment with Eu-conjugated α-Flag antibody. The data are expressed as relative fold activity as the mean ± SD (*n* = 3) compared to the control lacking Flag-ARM. ** *p* < 0.01 and “n.s.” indicates not significant. (**C**) The competitive inhibition curve of known inhibitory peptides was observed in a dose–response format. The IC_50_ values calculated for each competition assay are presented in the table box. The relative activities are represented as the mean ± SD (*n* = 3) compared with the DMSO control lacking the inhibitor. (**D**) The competition assay was performed by adding serial dilutions of non-tagged wt TAR RNA and its bulge-free mt form. Data are expressed as the mean ± SD (*n* = 3) compared to a control lacking an RNA competitor.

To evaluate the specificity of the TR-FRET assay, the mt Flag-ARM was incubated with wt 5′-Cy5-TAR RNA, which resulted in no signal similar to that shown in the reaction of the mt 5′-Cy5-TAR RNA coupled with wt Flag-ARM. In a competition assay, the TR-FRET signal was significantly decreased by a 10-fold excess of non-tagged wt ARM peptide as a competitor. In contrast, neither mt ARM nor BSA affected their interaction signals (Figure 3A). To test whether our assay is applicable for screening specific inhibitors of the Tat-TAR RNA interaction, inhibition assays were performed with known inhibitors of the Tat-TAR RNA interaction. As shown in Figure 3B, the ARM-TAR RNA interaction was significantly inhibited by the addition of known inhibitors with different efficiencies. A dose–response competition assay was conducted at inhibitor concentrations of 0–100 μM to determine their inhibitory curves in our assay. The IC_50_ values of these inhibitors obtained in our assay showed a trend similar to those of the previously described dissociation constants (K_d_) and IC_50_ values determined by their distinctive assays [35,36] (Figure 3C). Additionally, a dose-responsive competition assay using RNA fragments was performed to validate the specificity of the assay. A dose-dependent signal inhibition was observed by the addition of an increasing amount of non-tagged wt TAR RNA, but the inhibition was not significant with bulge-free TAR RNA (mt), even in 32-fold excess of analytes (1600 nM) (Figure 3D).

### 2.4. Suitability of the Assay in HTS

**Figure 4:**
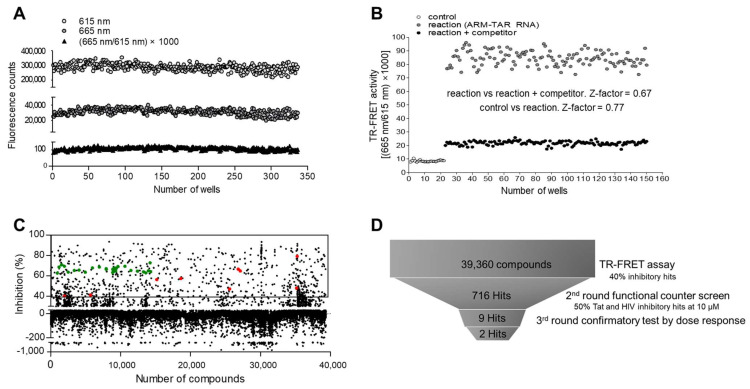
Consistency and suitability of the assay for high-throughput screening (HTS). (**A**) Fifty nanomoles of Flag-ARM and 5′-Cy5-TAR RNA were incubated in 320 wells of a 384-well microplate. Each fluorescent signal was measured, and the co-efficiency of variation (CV) of signal to background (665 nm/615 nm) was calculated as (standard deviation/mean) × 1000. (**B**) To validate the suitability for inhibitor screening, 50 nM of Flag-ARM and 5′-Cy5-TAR RNA were incubated with 500 nM of competitor peptide (non-Tagged ARM) in 140 wells. Twenty-five controls were incubated in the absence of Flag-ARM. The Z-factor values for each comparison are shown in the graph. The TR-FRET output signal is represented as the emission ratios of acceptor/donor (665 nm/615 nm) counts. (**C**) Pilot-scale screening of the 39,360-compound libraries using a TR-FRET assay. Two-hundred micromoles of compounds were incubated in a 384-well microplate with our TR-FERT assay mixture. The scatterplots represent percentage (%) inhibition compared with the DMSO treatment. The green and red dots indicate the positive experimental control (competitor ARM peptide) and nine hits obtained from the second-round screen, respectively. (**D**) A schematic funnel for screening inhibitors of the Tat-TAR RNA interaction.

To apply this assay to HTS using a 384-well microplate format, the consistency of the assay was evaluated. After excluding edge wells to avoid edge effects, 320 wells were used for the assessment of the assay. Two emissive fluorescent lights, representing the signal (665 nm) and background (615 nm), were consistently and sharply distinguished reciprocally. The experimental variation of each well was in the suitable range, with an 8.77% of the coefficient of variation (CV) for the signal (665 nm) to the background (615 nm) ratio (Figure 4A). To validate the suitability of our assay for screening inhibitors during HTS, two sets of repeatable 125 reactions were carried out in the presence or absence of 500 nM of competitor (ARM). Two signal bands from the reactions were clearly separated, and the Z-factor value was calculated to be 0.67, indicating the suitability of our assay for identifying potent inhibitory hit compounds during HTS. Furthermore, the Z-factor value of the reaction, compared with that of the negative control reaction lacking Flag-ARM (25 reactions), was 0.77 (Figure 4B). These data indicated that our assay is applicable to HTS using a large number of compound libraries to discover novel inhibitory molecules that disrupt the Tat-TAR RNA interaction.

### 2.5. Pilot Screen

**Figure 5:**
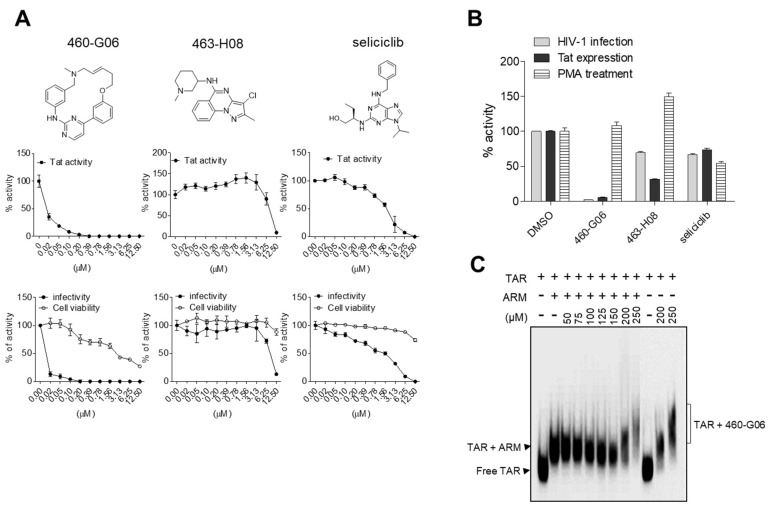
Biological activity of hit compounds on inhibition of HIV-1. (**A**) (Upper panel) Doxycycline (Dox)-inducible Tat-expressing bl-DTR cells cultured in 96-well plates treated with two-fold serial dilutions of each compound in the presence of Dox (50 ng/mL). At 24 h after treatment, the activity of firefly luciferase (F-Luc), indicating Tat activity, was determined as described in the Materials and Methods section. (Bottom panel) TZM-bl cells were treated with two-fold serial dilutions of the indicated compound and then infected with the HIV-1_NL4-3_ strain at a multiplicity of infection (MOI) of 1. At 24 h after infection, the viral infectivity and cell viability were determined as described in the Materials and Methods section. Seliciclib was used as a positive experimental control, and the data are presented as the mean ± SD (*n* = 3). The upper graphic data represent the chemical structure of hits. (**B**) 2 × 10^5^ TZM-bl cells growing in 6-well plates were infected with HIV-1 (MOI = 1), transfected with a Tat-expressing plasmid (2 μg) or treated with PMA (250 μg/mL) upon indicated compounds at indicated concentrations, respectively. At 24 h after treatment, LTR promoter activity was determined via a Bright-Glo Luciferase Assay Kit. The relative activities are represented as the mean ± SD (*n* = 3) compared with the DMSO vesicle. (**C**) The biotinylated TAR RNA (2 nM) was incubated with ARM peptide (100 nM) in the presence of increasing amounts of the hit compound and separated on a 3–12% native polyacrylamide gel. The mobility of biotinylated TAR RNA was detected by chemiluminescence.

To assess the applicability of our TR-FRET assay in identifying compounds against HIV-1 infection, a library of 39,360 compounds was screened using a contact-free automatic component transfer system at a fixed concentration of 200 μM. The inhibition percentage of each compound is presented as a scatter plot (Figure 4C). A total of 716 hits showing an inhibitory effect of over 40% were obtained in the primary TR-FRET screen with 500 nM of competitor (ARM), showing ~70% inhibition as a control (green dots). In the subsequent second-round functional assay based on a cellular system, nine compounds exhibited an inhibitory effect of more than 50% on both Tat-induced viral transcription and HIV-1 infectivity at a fixed concentration of 10 μM, which is relevant to medicinal applications (Figure 4D). In the third-round dose–response test, these hits were further evaluated at concentrations ranging from 0 to 12.5 μM. (Figure 5A and Figure S4). Of these, two compounds, 460-G06 [(*E*)-6-methyl-12-oxa-3,6-diaza-2(4,2)-pyrimidine-1,4(1,3)-dibenzenacyclododecaphan-8-ene] and 463-H08 [3-chloro-2-methyl-*N*-(1-methylpiperidin-3-yl)pyrazolo[1,5-a]quinazolin-5-amine], showed gradual inhibition of F-Luc activity, indicating Tat-induced viral transcription in a dose-dependent manner (Figure 5A upper panel and Table 1).

Next, the antiviral activity of these compounds was evaluated in TZM-bl cells infected with HIV-1_NL4-3_ in parallel with a cytotoxicity assay. Interestingly, the dose-responsive inhibitory effect of these compounds on HIV-1 infectivity was similar to that observed for Tat-induced transcription without severe cytotoxicity (Figure 5A). The IC_50_ values of 460-G06 and 463-H08 for HIV-1 infectivity were determined to be 0.013 and 8.055 μM, respectively. In particular, 460-G06 was more potent than seliciclib (IC_50_ value of 2.396 μM), a well-known inhibitor of Tat-induced viral transcription, which was used as a control (Table 1). The other seven compounds showed at least a 50% inhibitory effect on both Tat activity and HIV-1 infectivity at a concentration of 12.5 μM. Of the seven compounds, five (458-F08, 459-G07, 461-H04, 463-B11, and 464-A09) exhibited a gradual dose-responsive inhibitory effect on Tat activity, with a pattern similar to that of their antiviral effect. Additionally, 464-A09 showed a strong inhibitory effect on Tat and HIV-1 at 3.13 μM but showed sudden severe toxicity at 6.25 μM, indicating an off-targeting effect (Table 1 and Supplementary Figure S4). Two selected compounds inhibited the LTR-driven transcription in TZM-bl cells infected with HIV-1 or transfected with a Tat-expressing plasmid, but those effects were not observed under PMA treatment, which activates Tat-independent LTR transcription using cellular factors such as NF-κB (Figure 5B).

**Figure 6:**
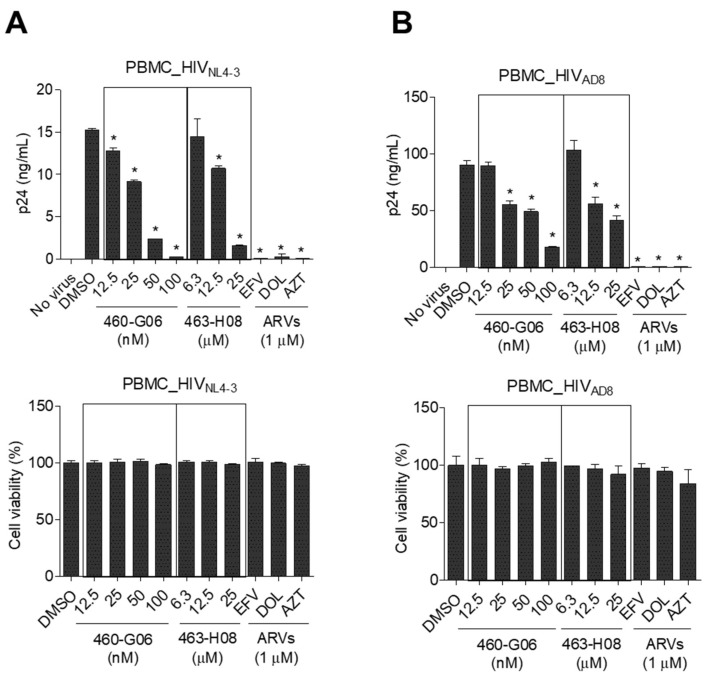
Phytohemagglutinin-preactivated peripheral blood mononuclear cells were infected with HIV-1_NL4-3_ (**A**) or HIV-1_AD8_ (**B**) at an MOI of 0.1 for 2 h and then treated with compounds at indicated concentrations for 72 h. The inhibitory effects of the compounds on viral replication and cell viability were determined as described in the Materials and Methods section. The data are expressed as the mean ± SD (*n* = 3). * *p* < 0.05 indicates a comparison with the cells treated with DMSO (vehicle). ARVs, anti-retroviral drugs; EFV, efavirenz; DOL, dolutegravir; AZT, azidothymidine.

The inhibitory effect of the potent hits on the interaction between ARM and TAR RNA was further validated using an RNA gel mobility shift assay. The most potent compound, 460-G06, directly bound to the TAR RNA and efficiently dissociated the ARM from TAR RNA. The K_d_ value of 460-G06 was determined to be 132 μM (Figure 5C). The 463-H08 exhibited relatively less dissociation effect (K_d_ = ~1000 μM). To determine the inhibitory effect of these compounds on the overall HIV-1 life cycle, HIV-1 replication assays were performed in peripheral blood mononuclear cells (PBMCs) infected with HIV-1_NL4-3_ (X4-tropic) and HIV-1_AD8_ (R5-tropic) in the presence or absence of these compounds (Figure 6A,B). The 460-G06 treatment inhibited viral replication in a dose-dependent manner at a concentration range of 12.5–100 nM without a severe decrease in cell viability, similar to their inhibitory effect on both viral infection and Tat activity. The 463-H08 treatment showed less inhibitory effect on viral replication, in accordance with its reduced potency against Tat and viral infection (Figure 6A,B).

## 3. Discussion

Although most screening approaches for discovering the inhibitor of HIV-1 Tat-mediated viral transcription have focused on disrupting the Tat-TAR RNA interaction, a specific drug disrupting this process has not been discovered yet. This is possibly because the identification of a potent compound in large-scale libraries using a complicated, insensitive, and laborious screening assay may be challenging.

Traditional biochemical/biophysical methods, including EMSA, fluorescence polarization (FP), and colorimetric assays, are widely used in screens that often require high concentrations, radioisotope labeling, and/or laborious steps such as multiple incubation/wash cycles. Recently, the homogenous FRET technology has been used to confirm the disruption of the Tat-TAR interaction by hits obtained from NMR docking screens [26]; however, it is often unsuitable for large-scale screens because of the relatively less distinguishable “true” signal caused by the simultaneous emissive noise from the donor and acceptor fluorophores [29]. Although the homogeneous FP assay is a powerful technique that is widely used to characterize biomolecular interactions and screen drugs, the assay often exhibits a high background signal owing to the local rotational effect (“propeller effect”) of the ligand and light scattering [37]. AlphaScreen (amplified luminescent proximity homogeneous assay; PerkinElmer) is an advanced bead-based method. Singlet oxygen is released from phthalocyanine (a photosensitizer) coated on donor beads and transferred to acceptor beads 200 nm in size. Although it has the particular advantage of detecting large biomolecules and/or weak interactions, it can be disadvantageous because it can potentially exhibit a lower inhibitory effect on the competitor than its actual potency [38]. Herein, we designed a simplified screening assay based on the ability of the TR-FRET technology to circumvent some problems associated with other techniques used to determine Tat-TAR RNA interactions. The TR-FRET assay using lanthanide cryptate is useful for determining the “true” signal by considering a short time delay between noise and the emitted signal from the acceptor.

The proximate distance (<10 nm) between the donor and acceptor fluorophores should be considered when designing a screening assay based on TR-FRET. Therefore, we attempted to minimize the two analytes, which might reveal the biological relevance of the Tat-TAR RNA interaction. The ARM of the Tat protein directly recognizes the bulge region on the TAR RNA [39], and the flanking residues of the core domain adjacent to the ARM increase the specificity of ARM binding to TAR RNA, similar to the full-length Tat protein [40]. Therefore, we used an ARM peptide that added several residues of the core region. The shortest stem-loop structure of TAR RNA (nt 19–43) contains a bulge that sufficiently binds to Tat and is necessary for Tat activity in vivo [9,10]. However, a slightly longer TAR RNA (nt 16–46) was used in our main experiments to reduce the structural instability of the stem loop as caused by unpaired bases on the bulge and mismatched bases on the stem (Figure 1C).

In our initial assay, the highest TR-FRET signal was exhibited by a reaction using biotinylated ARM recognized by Eu-StAV (Figure 1B). However, the TR-FRET assay using the biotin-StAV pair showed a weak inhibitory signal, even in the presence of high amounts of inhibitors disrupting the Tat-TAR RNA interaction (Supplementary Figure S1A). In addition, the reaction exhibited no decrease in the TR-FRET signal, even when a bulge-free mt 5′-Cy5-TAR RNA was used (Supplementary Figure S1B). The non-specific interaction signal between ARM and bulge-free mt TAR RNA was also detected in both assay systems using the 5′-biotinylated TAR RNA recognized by ULight-conjugated StAV as an acceptor (Supplementary Figure S2) and the 5′-biotinylated TAR RNA recognized by Eu-conjugated-StAV as a donor (Supplementary Figure S3). These results might be attributed to the fact that four biotinylated ARMs or biotinylated TAR RNAs captured by one Eu (or ULight)-conjugated StAV led to proximate abundance and that the strong avidity between biotin and StAV affected the conformational change of TAR RNA and/or ARM. Although the high avidity of biotin-StAV could serve an increased sensitivity for detecting weak interactions of certain biomolecules, it might not be useful in our TR-FRET assay to detect the Tat-TAR RNA interaction signal.

In the TR-FRET assay, the small proximity (<10 nm) between each fluorophore is a critical factor, and it is influenced by the size of analytes, tagged epitopes, and donor/acceptor-conjugated recognizers. Despite the similar size of ARMs tagged with different types of the epitope (HA, FITC, or Flag) in our study, the Flag-tagged ARM exhibited a more considerable TR-FRET signal. This might be due to the properties of the antibodies (such as specificity and/or affinity against the cognate tag) (Figure 1B). In an assay using Flag-ARM, the longest 5′-Cy5-TAR RNA of 59 nt showed the highest signal, which was not sufficiently decreased by the deletion of the bulge. These data revealed that the tertiary structure of TAR RNA might be attributed to the non-specific interaction of ARM or the proximity between Eu attached to antibody and Cy5 labeled to TAR RNA in homogenously mixed samples, leading to the interference of the TR-FRET “true” signal. Instead, a reduced non-specific signal was obtained using a short TAR RNA of 31 nt, but a complete abrogation of the non-specific signal was not observed (Figure 1D and Figure 3A), which was similar to the finding of a previous report that validated their SPA assay using the short bulge-free TAR RNA as a negative control [22]. Although these assays were focused on the central high affinity between Tat and TAR RNA, such slight non-specific signals might be due to the highly flexible structures of TAR RNA and Tat protein [26,41]. To determine the optimal conditions for the TR-FRET signal, a 2D-cross matrix titration of analytes and titrations of Eu-conjugated α-Flag antibody were performed. Especially, in a titration of Eu-conjugated α-Flag antibody, the maximum signal was achieved at 2.5 nM of the antibody, whereas a signal decrease was observed at > 2.5 nM, resulting in a “hook-effect” (Figure 2).

Our assay easily detected the specific inhibitory effects of known inhibitors, of which LK3 exhibited the greatest inhibitory effect on the ARM-TAR RNA interaction. The K_d_ value for the binding of LK3 to TAR RNA was determined to be 0.061 nM using a physical method [42], and the K_d_ values of L-22, -50, and -51 (as determined by EMSA) were 30, 1, and 5 nM, respectively [43]. The IC_50_ values of these compounds in the ARM-TAR RNA interaction, as determined by our assay, were 67.8 nM (LK3), 227.9 nM (L-22), 95.9 nM (L-50), and 276 nM (L-51), respectively. Although the K_d_ values differed from the IC_50_ values, the tendency of the K_d_ values from previous reports was mutually relevant to the IC_50_ values obtained in this study. Their K_d_ values also differed from their IC_50_ values obtained by biological experiments. This difference might be due to the methodological diversity of each experiment, such as the concentrations of analytes and the environments for the reaction.

In a pilot blind screen, our assay screened the primary 716 hits inhibiting the TR-FRET signal above ~40% from a compound library. At a rational concentration of 10 μM, nine hits satisfied the biological inhibition of both Tat and HIV-1, and two hits exhibiting dose-responsive inhibition of Tat activity were identified as novel inhibitory compounds against HIV-1 infection and replication with low cytotoxicity. The compound 460-G06 (known as SB1317 or TG02) showed a highly potent anti-HIV-1 activity but a gradual inhibitory effect on the cell viability in cancerous TZM-bl cells, indicating cytotoxicity at over 0.1 μM. The off-target effect might be related to its known biological activities, such as an anticancer effect caused by inhibiting cyclin-dependent kinases (CDKs), Janus kinase 2, and Fms-like tyrosine kinase-3 [44]. However, such cytotoxicity was not observed in primary PBMCs (Figure 5 and Figure 6). An activity linked to the CDKs inhibition of 460-G06 might also contribute to the Tat inhibition, apart from disrupting the Tat-TAR RNA interaction. Confirmation of this possibility may support the development of 460-G06 as an anti-HIV-1 agent. Although 463-H08 showed a lower inhibitory effect on HIV-1 infection than 460-G06, its biological activity was first proposed as a small molecule applicable to the development of anti-HIV-1 drugs. These hit compounds are under investigation by structure-activity relationship studies to develop potent lead compounds against HIV-1 infection. As our hit compounds showed common features such as positively charged amine groups and aromatic rings, which were proposed as linkers for binding to TAR RNA [13,17,26], a part of their antiviral activity may occur owing to the binding to TAR RNA, although this remains to be determined in a detailed structural study using NMR analysis. Additionally, the biological activity of these compounds, except for two compounds (460-G06 and 461-H04) known as anticancer agents [45], was first proposed here as an inhibitor against HIV-1. Recently, a potent TAR RNA binder, T0516-4834, was identified using an in silico docking technique. It inhibited HIV-1 infection with an IC_50_ = 0.2 µM and could disrupt the Tat-TAR RNA and Tat-CDK9 interactions [20]. T0516-4834 and most of our hit compounds contain aromatic rings connected with a positively charged amine group(s), similar to selicilib, which is known well as a CDK inhibitor. The structural similarities of these compounds might reveal the possibility that they inhibited the CDKs activity, resulting in a cooperative inhibitory effect on the overall Tat activity combined with inhibition of Tat-TAR RNA interaction.

Even though our assay exhibits a significant signal corresponding to an inhibitory effect on the Tat-TAR RNA interaction in vitro, the biological relevance of hit compounds screened by the assay may not always be ensured. This is because certain hit compounds are screened as false hits by interference with fluorophores and/or interaction between fluorophore-conjugated antibody and its epitope; are converted to non-functional structures by cellular metabolic enzymes; do not reach the target location upon the treatment of cells; and/or cause severe cytotoxicity. Therefore, the hit compounds obtained from the TR-FRET assay should be confirmed with a functional assay based on a cellular system sensitive to the inhibitory effect of Tat-mediated viral transcription [46]. Our TR-FRET assay combined with a functional assay showed good performance for rapidly identifying novel inhibitors of the Tat-TAR RNA interaction linked to the inhibition of HIV-1 infection.

## 4. Materials and Methods

### 4.1. Tat-Driven Peptides and TAR RNA Fragments

HIV-1_NL4-3_ Tat-derived peptides containing an ARM (RKKRRQRRR) were synthesized with each tag (FLAG, HA, FITC, and biotin) to probe donor or acceptor fluorophores at the N-terminus of each peptide (Peptron, Daejeon, Republic of Korea). A tag-free ARM and an mt ARM with five arginine (R) residues substituted with two aspartic acid (D) and three glutamic acid (E) residues were synthesized for use as controls (Table 2). Different lengths of 5′-Cy5- or biotin-tagged TAR RNA (HIV-1_NL4-3_) fragments were synthesized and purified by high-performance liquid chromatography as follows: TAR (59 nt, nt 1–59), TAR (36 nt, nt 13–48), and TAR (31 nt, nt 16–46). For the structural stability of TAR_31nt_, the adenosine (A) at position nt 17 and uracil (U) at position nt 46 were substituted with guanosine (G) and cytosine (C), respectively, as previously reported [47]. The mt TAR deleted with a three-thymidine bulge was used as an experimental negative control (Table 3 and Figure 1C).

### 4.2. Agents

Eu-conjugated StAV and anti-tag (Flag, HA, and FITC) antibodies were used to recognize biotinylated and tagged (Flag, HA, and FITC) ARMs as donor fluorophores. StAV-ULight was used to recognize 5′-biotinylated TAR RNA as an acceptor (Supplementary Figure S2). Eu-conjugated StAV (Eu-W8044-StAV) was used to recognize 5′-biotinylated TAR RNA as a donor, and ULight-conjugated anti-Flag and -FITC antibodies were used for the recognition of ARMs as acceptors (Supplementary Figure S3). All the recognizers conjugated with each fluorophore were purchased from PerkinElmer (Waltham, MA, USA). All TR-FRET experiments were carried out in 1× LANCE ultra assay buffer (PerkinElmer) supplemented with 40 units/mL of RNaseOUT (Invitrogen, Waltham, MA, USA). A known inhibitor of Tat-TAR RNA interaction, the dimeric LK3 peptide, was provided by Prof. Yan Lee (Seoul National University) [35], and cyclic peptides L-22, L-50, and L-51 were custom-synthesized from Peptron, as previously described [48]. A compound library of 39,360 chemicals was provided by the Korea Chemical Bank (www.chembank.org, accessed on 27 April 2020) of the Korea Research Institute of Chemical Technology. The hit compounds 460-G06 (known as SB1317) and 463-H08 were purchased commercially from TargetMol (Boston, MA, USA) and Wuxi Labnetwork (Shanghai, China).

### 4.3. TR-FRET Assay

#### 4.3.1. Cy5-TAR RNA:Tat (ARM)-Eu

The indicated concentrations of ARM and Cy5-TAR RNA fragments were incubated in 20 μL of 1× LANCE ultra assay buffer in 384-well microtiter plates with or without inhibitor (competitor) for 30 min at 20 °C in the dark. After incubation, 5 µL of the appropriate Eu-conjugated antibody capable of recognizing the tagged ARM peptide was added, followed by a 30-min incubation before the assay was measured. The reaction was performed in a final volume of 25 μL. Small volumes of the reaction solution were added automatically using Assist-Plus (Integra Bioscience, Zisers, Switzerland). After adding all the assay components, the plates were sealed with a clear cover. The ARM-TAR RNA interaction signal was measured in an EnSight multimode plate reader (PerkinElmer) using the LANCE protocol of the time-resolved fluorescence (TRF) mode in the Kaleido software (PerkinElmer), according to the manufacturer’s protocol. Briefly, the excitation light was directed to the donor (Eu) from the Xenon flash lamp at 320 nm selected by an optical filter, and then, an integration time of 100 μs and a delay time of 70 μs were provided to remove short-lived background signals; subsequently, two emission lights were detected at 615 nm for the donor (Eu) and 665 nm for the acceptor (Cy5). The TRF was measured from the top using an excitation filter and emission monochromators in a single measurement mode. The TR-FRET output signal was expressed as the emission ratio of acceptor/donor (665/615 nm × 1000) fluorescence counts. The fold activity of the TR-FRET signal for ARM-TAR RNA interaction was calculated compared to the control reaction lacking the ARM peptide.

#### 4.3.2. ULight-TAR RNA:Tat (ARM)-Eu

Each tagged ARM peptide and 5′-biotinylated TAR RNA were incubated in the dark for 30 min. Subsequently, StAV-ULight for TAR RNA (acceptor) and an appropriate Eu-conjugated antibody for tagged ARM (donor) were added and further incubated for 30 min in the dark. Signal measurement was performed as described above (Supplementary Figure S2).

#### 4.3.3. Eu-TAR RNA:Tat (ARM)-ULight

Each tagged ARM peptide and 5′-biotinylated TAR RNA were incubated, followed by incubation with ULight-conjugated antibodies recognizing tagged ARM and Eu-StAV (Eu-W8044-StAV). The TR-FRET signal was measured as described above (Supplementary Figure S3).

### 4.4. Assay for Tat-Mediated HIV-1 Transcription

The inhibitory effect of hit compounds on Tat-mediated transcription was determined using doxycycline (Dox)-inducible Tat-expressing TZM-bl cells that express the LTR-driven firefly luciferase (F-Luc) gene under the control of Tat upon Dox treatment, as described previously but with minor modifications [46]. In brief, 1 × 10^4^ of the cells were cultured in 96-well plates, treated with compounds at a concentration of 10 µM (in the second round of screening), and then treated with Dox (final concentration: 50 ng/mL). After 24 h, the inhibitory effect of the hit compounds on Tat activity was determined by measuring F-Luc activity using a Bright-Glo Luciferase Assay Kit (Promega, Madison, WI, USA). For the dose–response assay, cells cultured in 100 μL medium were treated with 50 μL of 1:2 serially diluted compounds at concentrations ranging from 0 to 12.5 µM and then treated with 50 μL of Dox (final concentration: 50 ng/mL). At 24 h post-treatment, the F-Luc activity was determined as described above. All luminescence assays were carried out in a SpectraMax Paradigm Microplate Reader (Molecular Devices, San Jose, CA, USA), and the data were expressed as a relative percentage (%) compared to the DMSO control (vehicle) in the presence of Dox. The experiments were performed in triplicate.

### 4.5. Antiviral Effects of the Compounds

To determine the inhibitory effect of hit compounds on HIV-1 infection, we used TZM-bl cells (also referred to as JC53BL-13), which express HIV-1 receptors and contain the LTR-driven F-Luc gene controlled by HIV-1 infection, as described previously but with some minor modifications [49]. Briefly, 1 × 10^4^ TZM-bl cells cultured on 96-well plates were treated in triplicate wells with 1:2 serially diluted compounds at concentrations ranging from 0 to 12.5 µM. Subsequently, the cells were infected with HIV-1_NL4-3_ virus obtained from the transfection of 293T cells at a multiplicity of infection (MOI) of 1. At 24 h after infection, the inhibitory effect of the hit compounds on infectivity was determined using the Bright-Glo Luciferase Assay Kit (Promega). The dose–response data are expressed as a relative percentage compared to the DMSO control (vehicle). The inhibitory effects of the hit compounds on HIV-1 replication were determined as previously described [50]. Briefly, 4 × 10^5^ PBMCs pre-activated with phytohemagglutinin M (PHA-M) were washed with fresh media and then infected with HIV-1_NL4-3_ or HIV-1_AD8_ at an MOI of 0.1 by spinoculation for 2 h. After infection, the cells were treated with hit compounds at the indicated concentrations at 3–5 days after treatment, and the inhibitory effect of hit compounds on viral replication was determined by the titration of virions from the cell supernatant using a p24 ALPHALISA™ Assay Kit (PerkinElmer) according to the manufacturer’s instructions. Cell viability was determined using resazurin-based PrestoBlue™ cell viability reagent (Thermo Fisher Scientific, Waltham, MA, USA) according to the manufacturer’s instructions. The data are presented as relative percentages compared to the DMSO control (vehicle). The experiments were performed in triplicate.

### 4.6. Gel Mobility-Shift Assay

To determine the inhibitory effect of hit compounds on the Tat (ARM)-TAR RNA interaction, a gel shift assay was carried out using the LightShift Chemiluminescent RNA EMSA Kit (Thermo Scientific) according to the manufacturer’s instructions, but with minor modifications suitable for our experiment. Briefly, 2 nM of 5′-biotinylated TAR RNA (nt 31) and 100 nM of ARM peptide were incubated with increasing amounts of hit compounds in the 1× REMSA binding buffer (10 mM HEPES pH 7.5, 10 mM MgCl_2_, 5 mM KCl, 1 mM DTT, and *v/v* 5 % glycerol) supplemented with 8 units/reaction of RNase OUT (Invitrogen) (final: 20 µL) at 20 °C for 20 min. Subsequently, the reaction was separated on a 3–12% native gradient PAGE gel. The RNA was then transferred to a nylon membrane to detect the mobility-shifted biotinylated TAR RNA using StAV-horseradish peroxidase conjugate. Chemiluminescence was detected using a GBox Chemi chemi-luminometer (Syngene, Fredrick, MD, USA).

### 4.7. Statistical Analysis

The Z-factor value was determined as previously described [51]. In brief, the Z-factor values for reaction vs. competitor were determined as follows: Z = 1 − (3 × STDEV_competitor_ + 3 × STDEV_reaction_)/(MEAN_competitor_ − MEAN_reaction_). The Z-factor for the control vs. reaction was determined as follows: Z = 1 − (3 × STDEV_reaction_ + 3 × STDEV_control_)/(MEAN_reaction_ − MEAN_control_). Graphical data were expressed as the mean ± SD (*n* = 3). Data were compared using Student’s *t*-test, and *p* < 0.05 was considered to indicate a significant difference. All statistical analyses were performed using GraphPad Prism 7.0.

## 5. Conclusions

Our results indicated that the TR-FRET assay that we established was suitable for identifying potent novel inhibitors of the Tat-TAR RNA interaction from libraries of chemical compounds. The novel compounds identified herein may serve as chemical scaffolds for developing a new class of HIV-1 drugs.

## Figures and Tables

**Table 1:** Dose responses of hit compounds on Tat and HIV-1 activity.

Hit	IC_50_^a^ of Tat Activity ^a^ (μM)	IC_50_ of Infectivity (μM)	CC_50_^b^ (μM)	SI ^c^
**460-G06**	**0.011 ± 0.001**	**0.013 ± 0.003**	**4.940 ± 0.440**	**380.000**
**463-H08**	**2.372 ± 0.160**	**8.055 ± 0.488**	**N.D.**	**>1.552**
458-D06	N.D.	N.D.	N.D.	-
458-F08	N.D.	1.286 ± 0.384	N.D.	>9.720
459-G07	3.929 ± 0.338	N.D.	N.D.	-
460-F06	3.204 ± 0.438	N.D.	N.D.	-
461-H04	10.063 ± 2.730	N.D.	N.D.	-
463-B11	6.816 ± 1.880	5.032 ± 0.310	N.D.	>2.484
464-A09	2.265 ± 0.518	2.029 ± 0.132	3.364 ± 0.160	1.645
Seliciclib	2.062 ± 0.409	2.396 ± 0.327	N.D.	>5.217

The dose responses of compounds were assessed in bl-DTR (for Tat activity) and TZM-bl cells (for infectivity and cytotoxicity), as described in the Materials and Methods section. ^a^ IC_50_: half-maximal inhibitory concentration; ^b^ CC_50_: concentration that reduces cell viability by 50%; ^c^ SI: selectivity index, defined as the ratio of IC_50_ to CC_50_; N.D.: not defined; Hits picked in the third-round assay are shown in bold.

**Table 2:** Tat-driven arginine-rich motif (ARM) peptides.

Name	Sequence
Biotin-Tat (ARM) (15 aa)	Biotin-GISYG**RKKRRQRRR**A
HA-Tat (ARM) (26 aa)	GAYPYDVPDYA-GISYG**RKKRRQRRR**A
Flag-Tat (ARM) (25 aa)	GADYKDDDDK-GISYG**RKKRRQRRR**A
FITC-Tat (ARM) (15 aa)	FITC-GISYG**RKKRRQRRR**A
Tagged-mutant Tat (ARM)	Tags-GISYGDKKDDQEERA
Tat (ARM) (15 aa)	GISYG**RKKRRQRRR**A

The Tat protein sequence was obtained from HIV-1_NL4-3_; The bold words indicate the arginine-rich motif (ARM); aa: amino acids.

**Table 3:** Information on trans-activating response (TAR) RNA fragments.

Name	Sequence	nt
TAR (59 nt)	5′-GGGUCUCUCUGGUUAGACCAGAUCUGAGCCUGGGAGCUCUCUGGCUAACUAGGGAACCC-3′	1–59
mt TAR (56 nt)	5′-GGGUCUCUCUGGUUAGACCAGA***GAGCCUGGGAGCUCUCUGGCUAACUAGGGAACCC-3′	1–59
TAR (36 nt)	5′-UUAGACCAGAUCUGAGCCUGGGAGCUCUCUGGCUAA-3′	13–48
mt TAR (33 nt)	5′-UUAGACCAGA***GAGCCUGGGAGCUCUCUGGCUAA-3′	13–48
TAR (31 nt)	5′-GGCCAGAUCUGAGCCUGGGAGCUCUCUGGCC-3′	16–46
mt TAR (28 nt)	5′-GGCCAGA***GAGCCUGGGAGCUCUCUGGCC-3′	16–46

A at nt 17 and C at nt 46 (bold and underlined) were substituted with G and C, respectively, to stabilize the stem-loop structure of the wt TAR RNA (31 nt) and mt TAR RNA (28 nt). Asterisks indicate deleted bases.

## Data Availability

All data presented in this study is contained within the article or supplementary material.

## Supplementary Materials

The supporting information can be downloaded at: https://www.mdpi.com/article/10.3390/ijms24119139/s1.
