# Supplementary material for: A Novel Time-Resolved Fluorescence Resonance Energy Transfer Assay for the Discovery of Small-Molecule Inhibitors of HIV-1 Tat-Regulated Transcription
Source: Int J Mol Sci. 2023 May 23;24(11):9139. doi: 10.3390/ijms24119139 (PMC10252837; doi:10.3390/ijms24119139)
Supplement: Supplementary file 1 [file ijms-24-09139-s001.zip › ijms-2404096-supplementary.pdf]

# Supplementary Figure S1

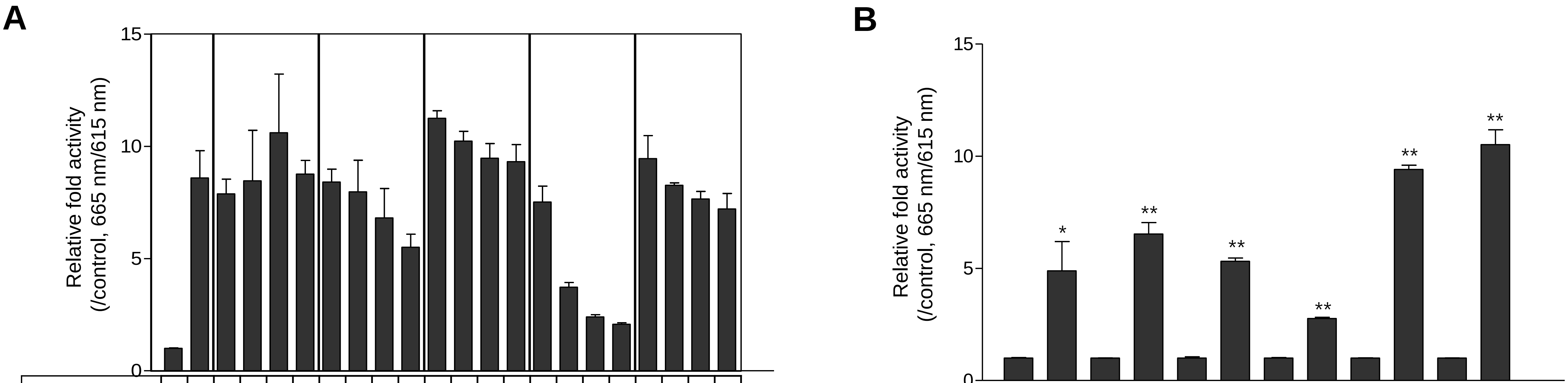


| | | | | | | | | | | | | | | | | | | | | | | |
|---|---|---|---|---|---|---|---|---|---|---|---|---|---|---|---|---|---|---|---|---|---|---|
| StAV-Eu | + | + | + | + | + | + | + | + | + | + | + | + | + | + | + | + | + | + | + | + | + | + |
| 5'-Cy5 TAR RNA | + | + | + | + | + | + | + | + | + | + | + | + | + | + | + | + | + | + | + | + | + | + |
| Bio-ARM | | + | + | + | + | + | + | + | + | + | + | + | + | + | + | + | + | + | + | + | + | + |
| Competitor | | | BSA | | | | ARM | | | | L-22 | | | | LK-3 | | | | L-50 | | | |
| (mmol/L) | | | 0.1 | 1 | 10 | 100 | 0.1 | 1 | 10 | 100 | 0.1 | 1 | 10 | 100 | 0.1 | 1 | 10 | 100 | 0.1 | 1 | 10 | 100 |

| | | | | | | | | | | | | |
|---|---|---|---|---|---|---|---|---|---|---|---|---|
| StAV-Eu | + | + | + | + | + | + | + | + | + | + | + | + |
| 5'-Cy5 TAR RNA (length, nt) | (59) *wt* | (59) *wt* | (56) *mt* | (56) *mt* | (36) *wt* | (36) *wt* | (33) *mt* | (33) *mt* | (31) *wt* | (31) *wt* | *(28)* *mt* | (28) *mt* |
| Biotin-ARM | - | + | - | + | - | + | - | + | - | + | - | + |

**Figure S1**. Evaluation of biotinylated Tat (ARM) for the TR-FRET assay (**A**) Twenty nanomoles of biotinylated-Tat (ARM) were incubated with 5′-Cy5-TAR RNA (31 nt) in the presence or absence of known inhibitors upon treatment with 5 nM Eu-StAV. (**B**) Twenty nanomoles of biotinylated Tat (ARM) were incubated with different lengths of wild type (*wt*) or cognate mutant (*mt*) 5′-Cy5-TAR RNAs upon treatment with 5 nM Eu-StAV. The data are expressed with relative fold activity of the TR-FRET signal as the mean ± SD (n = 3) compared with the control lacking biotinylated-Tat (ARM). *, P < 0.05 and **, P < 0.01 .

Supplementary Figure S2

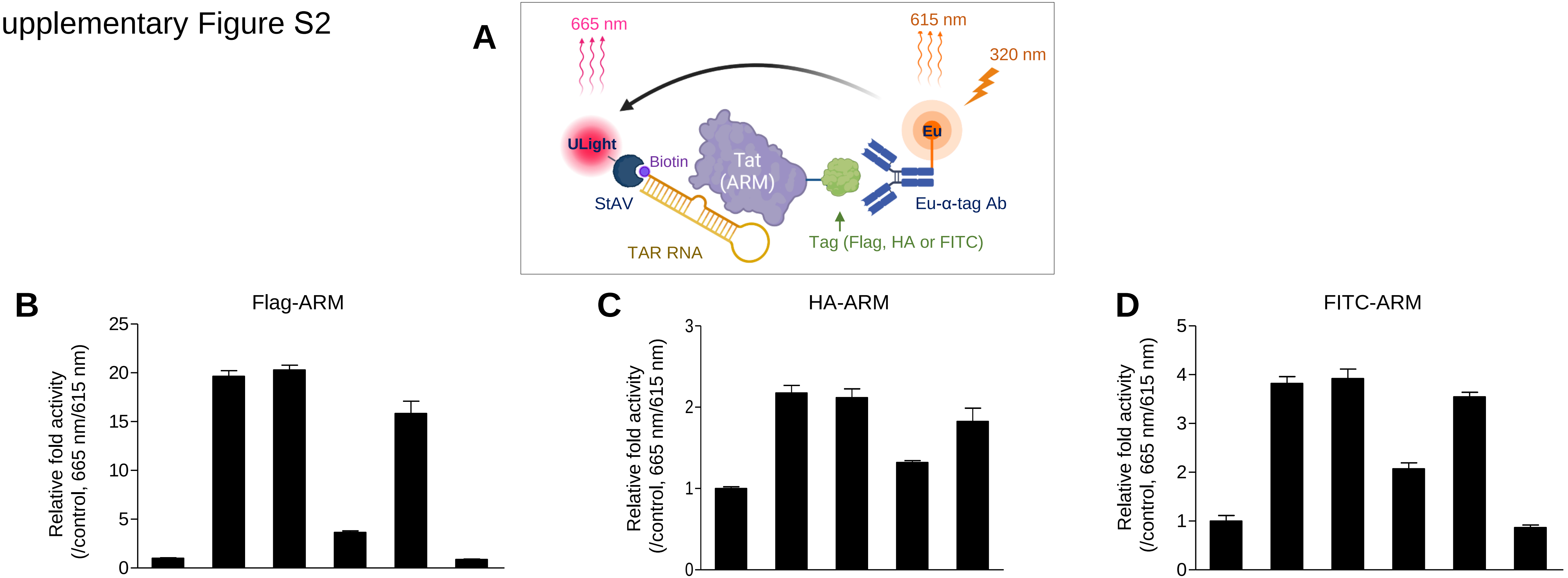


| | | | | | | |
|---|---|---|---|---|---|---|
| ULight-StAV | + | + | + | + | + | + |
| Biotin-TAR RNA | | + | + | + | *mt* | + |
| Eu-α-Flag | + | + | + | + | + | + |
| Flag-ARM | + | + | + | + | + | *mt* |
| Competitor | | | BSA | ARM | | |

| | | | | | |
|---|---|---|---|---|---|
| ULight-StAV | + | + | + | + | + |
| Biotin-TAR RNA | | + | + | + | *mt* |
| Eu-α-HA | + | + | + | + | + |
| HA-ARM | + | + | + | + | + |
| Competitor | | | BSA | ARM | |

| | | | | | | |
|---|---|---|---|---|---|---|
| ULight-StAV | + | + | + | + | + | + |
| Biotin-TAR RNA | | + | + | + | *mt* | + |
| Eu-α-FITC | + | + | + | + | + | + |
| FITC-ARM | + | + | + | + | + | *mt* |
| Competitor | | | BSA | ARM | | |

**Figure S3.** ULight-TAR RNA:Tat (ARM)-Eu **(A)** Schematic illustration of the assay based on TR-FRET to detect the interaction between the biotinylated TAR RNA and tagged-Tat (ARM) using StAV-ULight and Eu-conjugated *α*-tag antibody **(B-D)** Fifty nanomoles of wild or mutant (*mt*) type of 5′-biotinylated TAR RNA (31 nt) was incubated with 50 nM each of Flag (B), HA (C) and FITC (D)-tagged Tat (ARM)) upon 2.5 nM of the donor (Eu-conjugated *α*-tag Ab) and acceptor (ULight-StAV) fluorophores in the presence or absence of ARM as a competitor. The data are expressed as relative fold activity of the TR-FRET signal as the mean ±SD (n =3) compared with control lacking tagged-Tat (ARM).

# Supplementary Figure S3

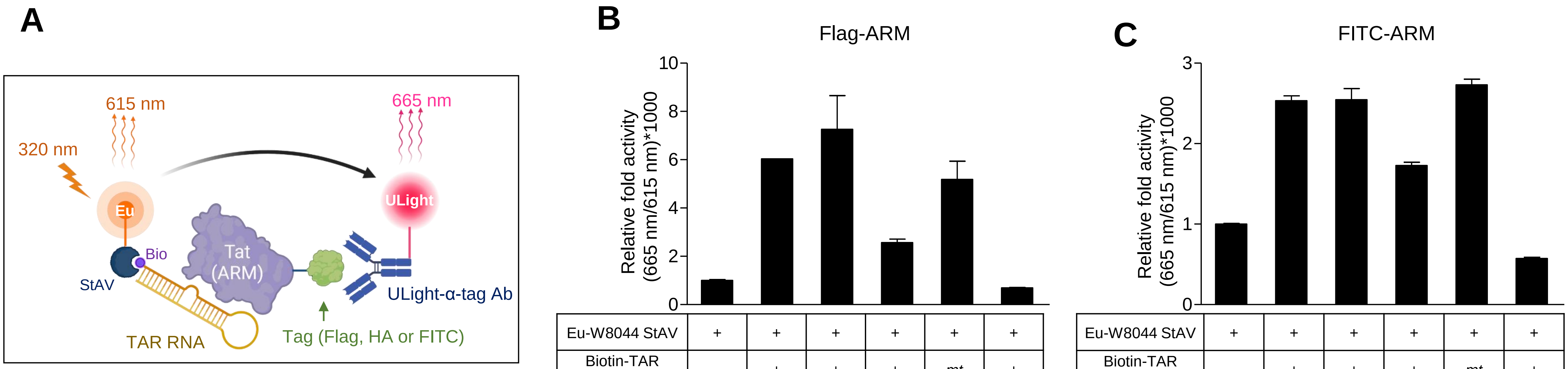


**B (Flag-ARM)**

| | | | | | | |
|---|---|---|---|---|---|---|
| Eu-W8044 StAV | + | + | + | + | + | + |
| Biotin-TAR RNA | | + | + | + | *mt* | + |
| Flag-ARM | + | + | + | + | + | *mt* |
| ULight-α-FLAG | + | + | + | + | + | + |
| Competitor | - | - | BSA | ARM | - | - |

**C (FITC-ARM)**

| | | | | | | |
|---|---|---|---|---|---|---|
| Eu-W8044 StAV | + | + | + | + | + | + |
| Biotin-TAR RNA | | + | + | + | *mt* | + |
| FITC-ARM | + | + | + | + | + | *mt* |
| ULight-α-FITC | + | + | + | + | + | + |
| Competitor | - | - | BSA | ARM | - | - |

**Figure S3.** Eu-TAR RNA:Tat (ARM)-ULight **(A)** Schematic illustration of the assay based on TR-FRET to detect the interaction between biotinylated TAR RNA and tagged Tat (ARM) using Eu-StAV and ULight-conjugated α-tag antibody **(B-C)** Fifty nanomoles of wild or mutant (*mt*) type of 5′ biotinlyated TAR RNA (31 nt) was incubated with 50 nM of wild or mutant (*mt*) type of Flag (B) and FITC (C)-tagged Tat (ARM) upon 2.5 nM of donor (Eu-StAV) and acceptor (ULight-conjugated α-tag Ab) fluorophores in the presence or absence of ARM as a competitor. The data are expressed as relative fold activity of the TR-FRET signal as the mean ±SD (n =3) compared with control lacking tagged-Tat (ARM).

# Supplementary Figure S4

| | | | | | | | |
|---|---|---|---|---|---|---|---|
| Name | 2-Methyl-*N*-(4-(2-(5-morpholinopyridin-2-ylamino)-2-oxoethyl)thiazol-2-yl)furan-3-carboxamide | 2-(2-(8-Chloro-2,3-dihydrobenzo[*b*][1,4]dioxin-6-yl)vinyl)-4,5,6,7-tetrahydrobenzo[*d*]thiazole-4-carboxylic acid | 5-Methyl-*N*-(5-(piperidin-4-yl)thiazol-2-yl)-3-(pyridin-2-yl)isoxazole-4-carboxamide | 4-(3-(3-(2,4-Difluorobenzyl)-2-imino-2,3-dihydro-1*H*-benzo[*d*]imidazol-1-yl)-2-hydroxypropyl)-3-(4-(trifluoromethyl)phenyl)-1,2,4-oxadiazol-5(4*H*)-one | *N*-(3-Methyl-2-(4-methylpiperazin-1-yl)butyl)-2-(thiophen-3-yl)quinazolin-4-amine | 5-Amino-3-(3-(5-*p*-tolylthieno[2,3-*d*]pyrimidin-4-ylamino)propyl)-1*H*-pyrazole-4-carbonitrile | 6,11-Dioxo-3-(3-(piperidin-1-yl)propyl)-5-(*p*-tolylamino)-6,11-dihydro-3*H*-anthra[1,2-*d*][1,2,3]triazole 2-oxide |
| Structure | 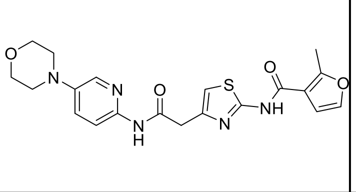 | 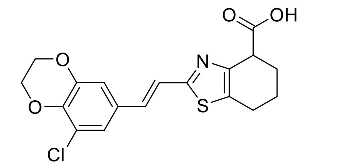 | 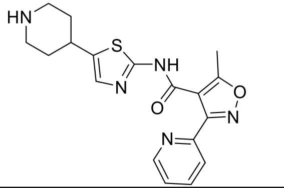 | 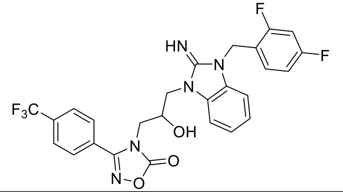 | 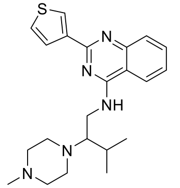 | 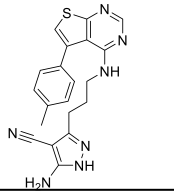 | 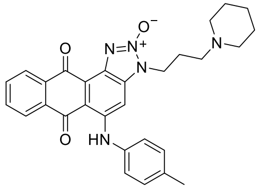 |

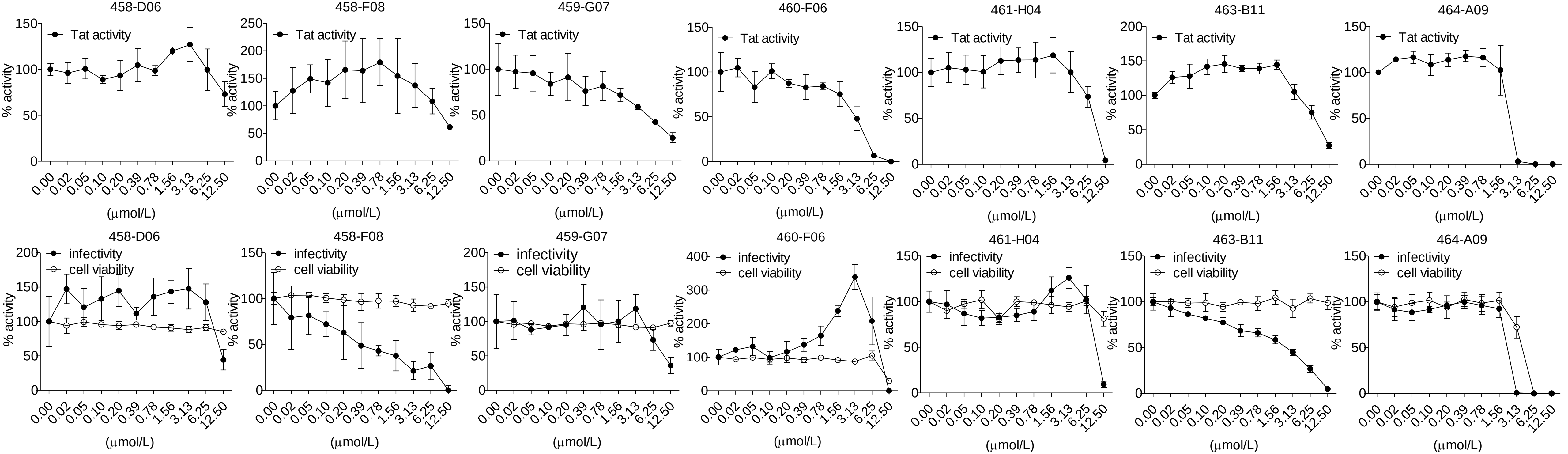


**Figure S4.** Dose-responsive inhibitory effects of hit compounds on Tat and HIV-1 infection. (Upper panel) bl-DTR cells cultured in 96-well plates treated with two-fold serial dilutions of each compound in the presence of doxycycline. At 24 h after treatment, the activity of firefly luciferase (F-Luc) indicating Tat activity was determined as described in the Materials and Methods section. (Bottom panel) TZM-bl cells were treated with two-fold serial dilutions of the indicated compound and then infected with the HIV-1$_{NL4-3}$ strain at an MOI of 1. The viral infectivity and cell viability were determined as described in the Materials and Methods section. The upper graphic data represent the chemical name and structure of hits.
